# Infectious endocarditis caused by *Bartonella henselae* associated with infected pets: two case reports

**DOI:** 10.1186/s13256-023-03839-8

**Published:** 2023-04-19

**Authors:** Jonathan Gonçalves-Oliveira, Paulo Vieira Damasco, Matheus Ribeiro da S. Assis, Dominique E. Freitas, Adonai Alvino Pessoa Junior, Luiza S. de Sousa, Nicollas G. Rodrigues, Paula H. Damasco, Pedro F. Ribeiro, Giovanna F. Barbosa, Namir S. Moreira, Jeferson F. Guedes, Henrique M. da Rocha Coutinho, Kalil Madi, Elba R. Sampaio de Lemos

**Affiliations:** 1 Laboratório de Hantaviroses E Rickettsioses, Instituto Oswaldo Cruz (IOC/FIOCRUZ), Manguinhos, Rio de Janeiro, RJ Brazil; 2 Faculdade de Ciências Médicas, Time de Endocardite Do Hospital Universitário Pedro Ernesto, Universidade do Estado do Rio de Janeiro (HUPE/UERJ), Rio de Janeiro, RJ Brazil; 3 Escola de Medicina e Cirurgia, Departamento de Doenças Infecciosas e Parasitárias, Universidade do Federal do Estado do Rio de Janeiro (DIP/UNIRIO), Rio de Janeiro, RJ Brazil; 4 Faculdade de Veterinária - Anhanguera, Niterói, RJ Brazil; 5 Serviço de Cardiologia e Ecocardiografia, Hospital Municipal Miguel Couto (HMMC-RJ), Leblon, Rio de Janeiro, RJ Brazil; 6 Diretoria Técnica do Círculo Brasileiro de Patologia, Vila Isabel, Rio de Janeiro, RJ Brazil

**Keywords:** Endocarditis, *Bartonella*, Pets, One Health, Case report

## Abstract

**Background:**

Blood culture-negative infective endocarditis is a potentially severe disease that can be associated with infectious agents such as *Bartonella* spp., *Coxiella burnetti*, *Tropheryma whipplei*, and some fungi.

**Case presentation:**

Reported here are two cases of blood culture-negative infective endocarditis in patients with severe aortic and mitral regurgitation in Brazil; the first case is a 47-year-old white man and the second is a 62-year-old white woman. *Bartonella henselae* deoxyribonucleic acid was detectable in the blood samples and cardiac valve with vegetation paraffin-fixed tissue samples. Additionally, an investigation was carried out on patients’ pets, within the context of One Health, and serum samples collected from cats and dogs were reactive by indirect immunofluorescence assay.

**Conclusions:**

Even though the frequency of bartonellosis in Brazil is unknown, physicians should be aware of the possibility of blood culture-negative infective endocarditis caused by *Bartonella*, particularly in patients with weight loss, kidney changes, and epidemiological history for domestic animals.

**Supplementary Information:**

The online version contains supplementary material available at 10.1186/s13256-023-03839-8.

## Background

Infective endocarditis (IE) is a life-threatening systemic infectious disease in which a multidisciplinary group of specialists is required for patient treatment and follow-up. *Viridans streptococci*, *Streptococcus bovis*, *Haemophilus* spp., *Aggregatibacter* spp., *Cardiobacterium hominis*, *Eikenella corrodens*, and *Kingella* spp. (HACEK) group, *Staphylococcus aureus*, or community-acquired enterococci are classic typical microorganisms causing IE, an infection associated with the proliferation of microorganisms on the endocardium [1]. Vegetation is the prototypic lesion of IE, which can be identified by a mass of platelets, fibrin, and microcololia of bacteria, fungi, or other germs and scant inflammatory cells. The vegetation of IE is located on the mural endocardium and analogous processes can occur in intracardiac devices, arteriovenous shunts, or coarctation of the aorta [2]. The risk factors of IE involve patients with predisposing heart conditions and comorbidities, including intravenous drug use, among other factors [3]. The diagnosis of IE is established according to the modified Duke criteria [1, 4], which include predisposing heart condition or intravenous drug use, symptoms such as fever ≥ 38 °C, vascular phenomena, immunologic phenomena, and serologic evidence of active infection consistent with IE or positive blood culture but not meeting major criteria [1, 2, 4, 5]. Major modified Duke criteria, in addition to including the persistently positive blood culture and evidence of endocardial involvement, now include positive serology for *Coxiella burnetii* and *Bartonella* spp.

At least two separated positive blood cultures samples, with typical microorganisms, are required to meet major microbiological criteria. Gram-negative bacillus such as *Bartonella* spp., *C*. *burnetti*, and *Tropheryma whipplei*, some fungi such as *Aspergillus* spp. and *Histoplasma* must be searched when facing blood culture-negative infective endocarditis (BCNIE), since these etiological agents are harder to be cultivated *in vitro* [6–9]. Specifically, *C*. *burnetti*, *Bartonella quintana*, and *Bartonella henselae* are not easy to isolate in blood cultures, and classic Duke criteria do not identify patients with IE.

In this context, serological and molecular methods have become an important tool in the detection of *C*. *burnetti* and *Bartonella* spp. in the last decades in Brazil. The genus *Bartonella* is recognized as the second most frequent etiological agent in HACEK group, non-HACEK groups, and BCNIE. *B*. *henselae* is the most prevalent of the 14 species of associated with *Bartonella* endocarditis. This species is also known to be the main cause of cat scratch disease (CSD), a typical zoonotic disease in Brazil [10]. Although domestic cats and their ectoparasites are often found as hosts of *B*. *henselae* [11–13], dogs have also been reported to host this species [14, 15].

The risk is not limited to clinical cases in humans; dogs and cats can also develop cardiac complications related to infection by *Bartonella* [10, 16–20]. Bartonellosis is a disease of medical and veterinary importance, so One Health becomes a strategy to approach this epidemiological context. The One Health approach integrates a multidisciplinary team of researchers from different areas of knowledge to cooperate in the diagnosis, prevention, treatment, and mitigation of infection diseases [21]. The most common health professionals are typically physicians, veterinarians, and biologists linking human, animal, and environmental health [21]. Thus, the One Health epidemiological approach, such as in this study, could make more data available in the IE scenario associated with zoonoses [19].

In Brazil, where most cases of endocarditis are found in the Southeast region, we still have few epidemiological data on *Bartonella* endocarditis, although we have been working on retrospective studies since 2006 [5, 8, 9, 22–24]. The aim of this report is to show two cases of *B*. *henselae* endocarditis associated with *Bartonella*-infected domestic animals during the COVID-19 pandemic in Rio de Janeiro, in the southeast region of Brazil. We considered the modified Duke criteria to establish the diagnosis of IE caused by *Bartonella*, including the presence of titer Immunoglobulin type G (IgG) antibodies against *Bartonella* spp. ≥ 800 of dilution and a positive result of molecular testing obtained from cardiac valve and blood samples.

## Case presentation

### Patient 1

**Fig. 1:**
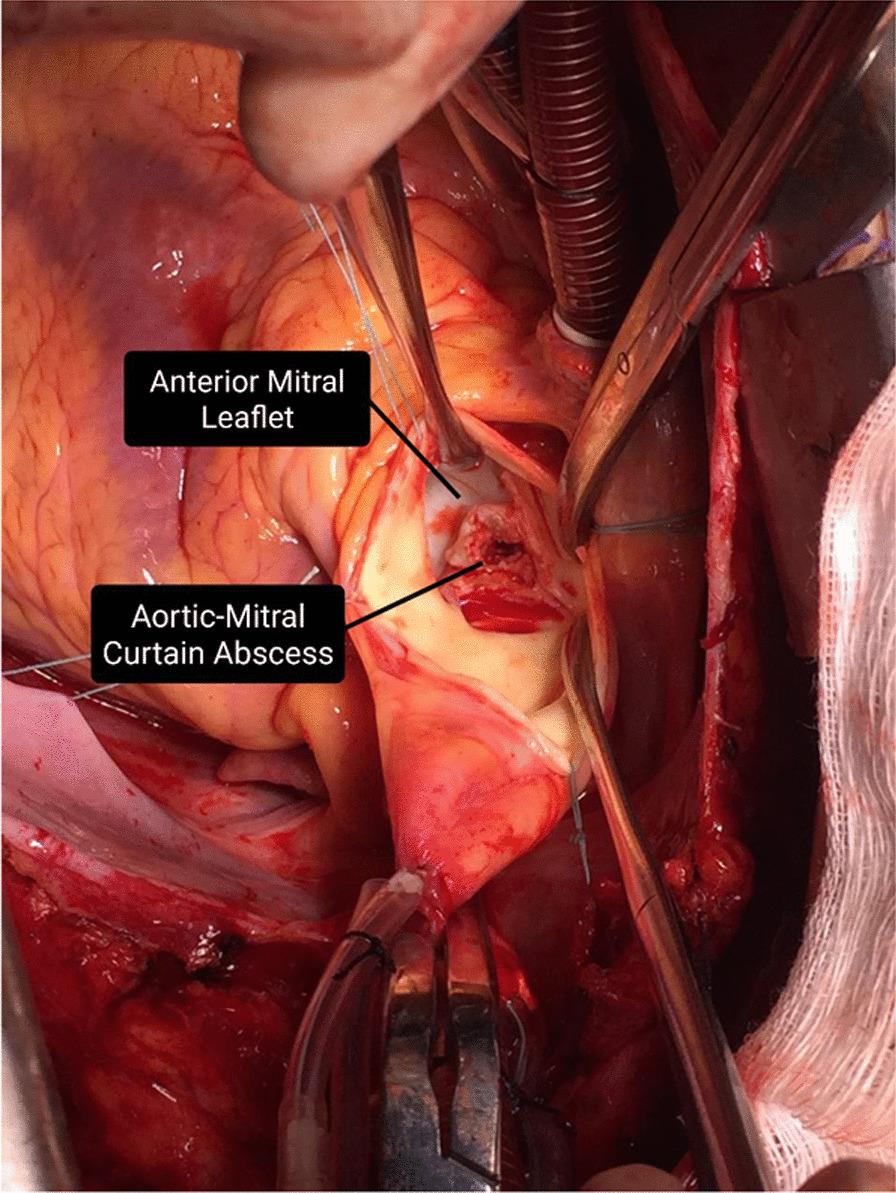
Aortic–mitral curtain abscess visualized after resection of aortic valves: patient 1 with blood culture-negative infective endocarditis. The black arrows correspond to the pointed structures described

**Fig. 2:**
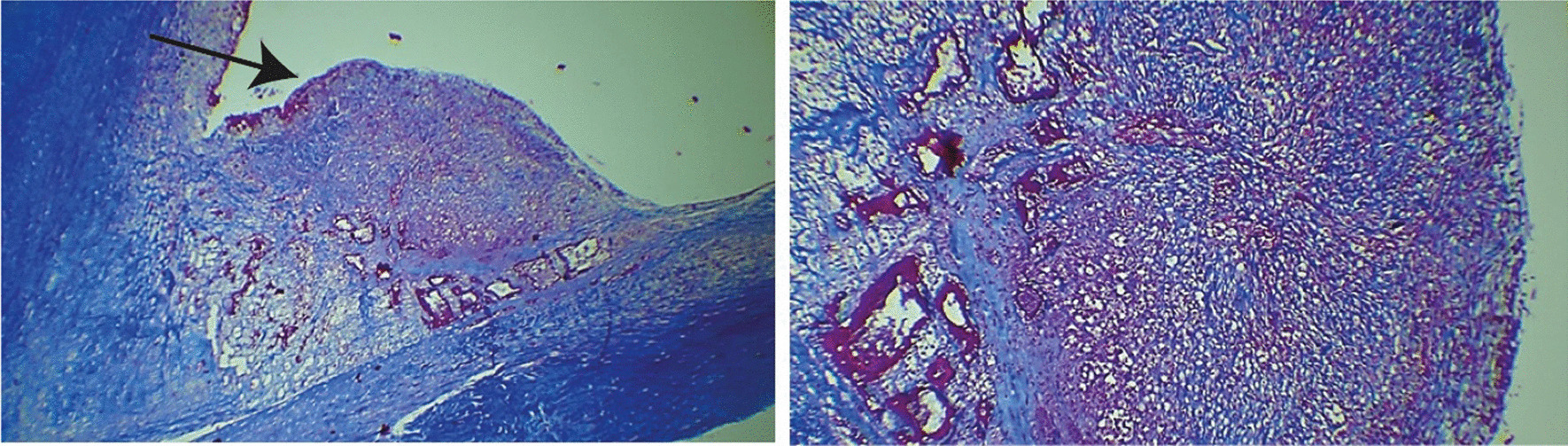
Left - Masson trichrome staining micrographs showing an inflammatory nodule (pointed by the arrow) in the valve vegetation of patient 1 with blood culture-negative infective endocarditis. Right – Enlarged and detailed left micrograph, showing the nodule with fibrosis, vascular neoformation, and chronic inflammatory infiltrate

A 47-year-old white man was admitted to Pedro Ernesto University Hospital of the State of Rio de Janeiro (HUPE-UERJ) with a history of tachycardia palpitations, atrial fibrillation associated with recurrent episodes of fever, and weight loss of 3 kg in the last 40 days. At admission, he had a mild acute kidney injury, with hematuria and no proteinuria. The echocardiogram showed severe mitral and aortic stenosis associated with rheumatic valvular disease and aortic valve vegetation, with normal left and right ventricular systolic function. He was febrile and had high levels of C-reactive protein (CRP), erythrocyte sedimentation rate (ESR), and rheumatoid factor (RF). As comorbidities, he had tooth infections and recent untreated nephrolithiasis. Three sets of blood culture samples were collected at admission, and they were all negative. Antibiotic therapy was withheld since there was no clear evidence of vegetation seen on transesophageal echocardiography (TEE). However, there was a severe aortic insufficiency with clear evidence of rheumatic valve degeneration, and due to the risk of imminent death, the patient was referred for a surgical procedure. The entire surgical procedure was performed using transesophageal echocardiography and an aortic valve was evidenced with fusion of its cusps and friable additive image, compatible with vegetation caused by infective endocarditis. The valve was resected and it was possible to see an abscess affecting the mitral aortic curtain. The mitral valve was also rheumatic and dysfunctional. The two valves were replaced uneventfully and the extracted material was sent for analysis. The collected samples were analyzed by the pathologist in the operating room, confirming the diagnostic suspicion (Fig. 1). Samples were collected and fixed in formalin and embedded in paraffin for histopathological and molecular studies. The mitral and aortic valves were replaced, and the left auricle was clipped. Histological examination of the valve vegetation, preserved in 10% formalin, demonstrated the presence of inflammatory infiltrate associated with mononuclear cells and macrophages, vascular neoformation, and fibrosis (Fig. 2). After the intraoperative impression, treatment for subacute endocarditis was started with gentamicin and ampicillin.

Acute-phase and convalescent-phase serum samples were evaluated by an indirect immunofluorescence assay (IFA) for antibodies anti-*Bartonella* considering a cut-off titer of 64 (Additional file 1: Methods S1). The serum sample collected on first day of hospital admission, before antibiotic treatment therapy, was IFA reactive, with titer of 8192. Three other sequential serum samples were analyzed and were also reactive: (i) after antibiotic treatment, before the surgical procedure, titer of 16,384, (ii) after cardiac surgery, titer of 32,768, and (iii) at his house, titer of 2048 (Additional file 1: Table S1).

In relation to polymerase chain reaction (PCR) assay, despite blood, serum, and plasma samples being negative, the PCR assay on the paraffin-fixed tissue samples targeting the *gltA*, *groEL*, and *htrA* genes were evaluated by routine of the National Reference Laboratory for Rickettsioses (NRLR), Oswaldo Cruz Institute (IOC), and Oswaldo Cruz Foundation (FIOCRUZ) and segments of *Bartonella* genes were amplified (Additional file 1: Methods S1). The DNA sequence of *gltA* was similar 99.84% to KX024515 Uncultured *Bartonella* of domestic cat blood samples (*B*. *henselae*), the DNA sequence of *groEL* was similar 100% to HQ704721 *B*. *henselae* isolate of feral cat blood, and the DNA sequence of *htrA* was similar 100% to CP020742, *B*. *henselae* strain Houston-I (Additional file 1: Table S2).

**Fig. 3:**
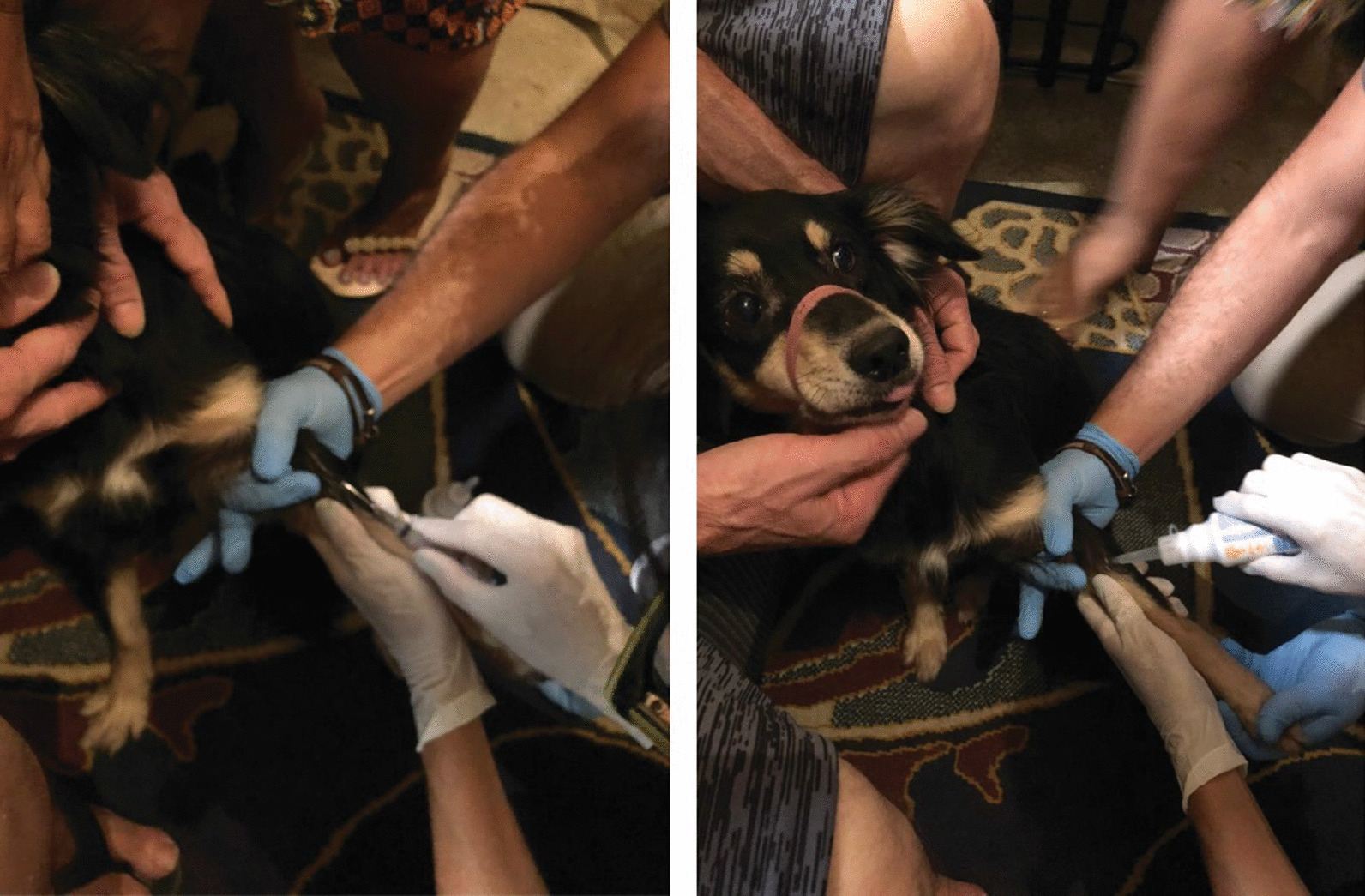
Veterinarian professional collecting blood sample from a *Bartonella*-serorreactive dog

The patient had two adult dogs, and one of them slept with him and his wife. Serum samples collected from the patient’s wife were nonreactive. Dogs were assessed by veterinarians for the presence of ectoparasites, general aspects of health, and living conditions in the home environment (Fig. 3). Blood serum samples were collected from these animals and serum samples from one of his dogs was reactive with titer of 128. The patient subsequently improved and was discharged for outpatient follow-up.

### Patient 2

Patient 2 is a 62-year-old white woman with a history of chronic renal disease, anemia, and reported loss of 10 kg in the last 9 months. She was admitted to the Pedro Ernesto University Hospital of the State of Rio de Janeiro (HUPE-UERJ) to explore the cause of consumptive syndrome. She had undergone a mitral valve prothesis replacement with a biological prosthesis 2 years before due to complications from rheumatic heart disease. Echocardiography revealed a dysfunctional biological mitral valve associated with rheumatic valve disease. The patient also had acute on chronic renal failure with hematuria, severe anemia, splenomegaly, paratracheal lymphadenomegaly, and arterial hypertension, associated with high levels of C-reactive protein (CRP), erythrocyte sedimentation rate (ESR), and rheumatoid factor (RF). During hospitalization, kidney biopsy showed glomerulonephritis with crescents, tubular atrophy, and interstitial fibrosis. Blood culture samples were all negative and the patient was treated with ceftriaxone and doxycycline. She improved and was discharged for outpatient follow-up.

Considering a possible diagnosis of IE, according to modified Duke criteria, three minor criteria blood samples were sent to the National Reference Laboratory for Rickettsioses (NRLR) to research etiologic agents associated with BCNIE (Additional file 1: Methods S1). Two serum samples were reactive to *Bartonella* IgG-antibodies: (i) with titers of 8192, without antibiotic treatment, and (ii) with titers of 512, subsequently after antibiotic treatment (Additional file 1: Table S1). *Bartonella* spp. DNA was detectable in the blood by *htrA* primers and this sequence of *htrA* was similar 99.76% to CP020742 *B*. *henselae* strain Houston-I (Additional file 1: Table S2).

**Fig. 4:**
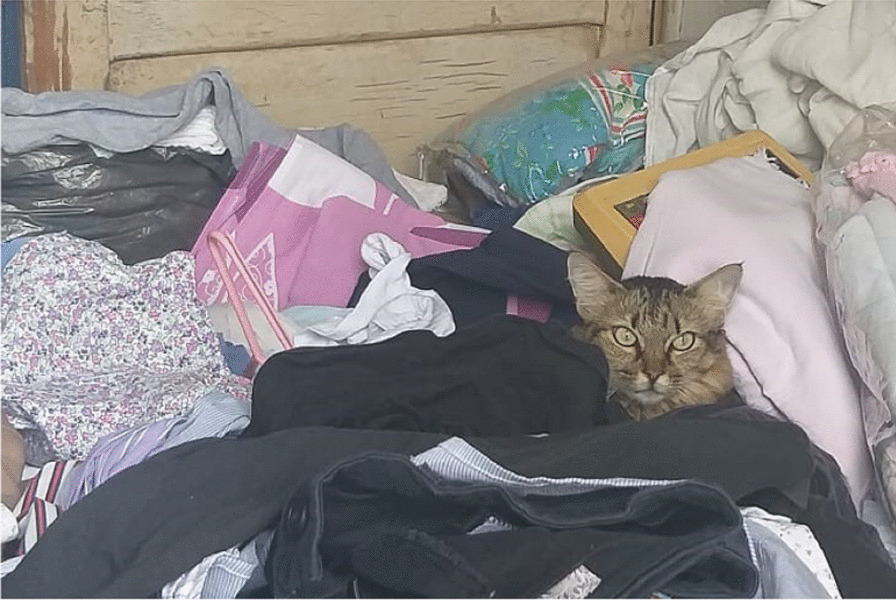
*Bartonella*-serorreactive cat in patient 2’s house, showing the unhealthy living conditions

During home visits, three cats were assessed by veterinarians for the presence of ectoparasites, general aspects of health, and living conditions in the home environment (Fig. 4). Subsequently, serum samples were collected from these animals, and all were reactive with titers of 128 (Additional file 1: Table S1).

## Discussion and conclusions

*Bartonella* infections occur worldwide and can be associated with blood culture-negative infective endocarditis (BCNIE). Since the first publication of infective endocarditis due to *Bartonella* spp. confirmed by serologic test in Brazil, few cases of this endocarditis infection have been reported, although cases of cat scratch disease (CSD) are often reported, especially in the southeast Brazilian region [8].

On the basis of clinical and diagnostic protocols in this case report, both patients met the evaluation criteria for infective endocarditis associated with blood culture-negative, a heart disease infection often severe and difficult to diagnose. In view of the inconclusive microbiological research with blood cultures, serum samples collected from the two patients and cardiac valve tissue sample (patient 1) were analyzed by both serological and molecular tests and the results confirmed infection by *B*. *henselae*. Often the *Bartonella* diagnosis is determined using serological tests, mainly by IFA. In this study, the patients presented high IgG-antibodies titers to *Bartonella* spp., a fact that allows the confirmation of BCNIE caused by this zoonotic agent exclusively by serological testing, since the IFA results with IgG titers ≥ 1:800 have a sensitivity of 90% and specificity of 99%.

Subsequent molecular analysis performed on blood and cardiac tissue (mitral valve) sample collected from patients allowed for the identification of the *B*. *henselae* species as the causative agent of BCNIE. Since 2009, the National Reference Laboratory for Rickettsioses/Ministry of Health (NRLR), Laboratory of Hantaviruses and Rickettsiosis, Oswaldo Cruz Institute, and FIOCRUZ, have been collaborating with our IE team at our teaching hospital. It is the first time we have *B*. *henselae* endocarditis in our series of 110 cases of IE defined by modified Duke criteria in Rio de Janeiro, Brazil. The incidence of BCNIE in this Brazilian cohort was 10.9%, although the frequency of BCNIE in review of IE in low- and middle-income countries during the years 2002–2017 ranged from 10.8–69.1% [5].

These *Bartonella* IE cases are a classic example of a zoonosis where a One Health approach must be applied [19]. After the confirmation of endocarditis caused by *B*. *henselae*, a multidisciplinary team carried out visits at the homes of both patients. The participation of a veterinarian allowed for the handling of domestic animals, the collection of blood samples, and the search for ectoparasites in these animals. From the epidemiological inquiry, both patients maintained very close contact with the animals, living with the pets until bedtime.

Serological investigation of domestic animals showed IgG reactivity to *Bartonella* spp. in the patients’ pets. Information regarding intimate contact with these pets, as well as our serological results, reinforce the likelihood of contagion through this human–pet relationship. It is interesting to consider that during the isolation caused by the SARS-CoV-2 pandemic, this contact between humans and their pets increased, which may have facilitated exposure to *B*. *henselae*. In this context, the presence of ectoparasites and the possibility of injury caused by scratches can facilitate the inoculation of this agent in humans [11, 26, 27].

Despite evidence of circulation of *Bartonella* spp. having already been reported in other studies from Rio de Janeiro [9, 28], in these case reports we indicate which bacterial species was found in these patients, which facilitates the understanding of the epidemiological aspects and the investigation of human cases from a One Health initiative [19]. The formation of a multidisciplinary team is the most effective way to identify zoonotic agents such as *Bartonella* that cause infections in humans and animals, also considering the non-specificity of this bacteria genus, which can be found in humans as well as in domestic and wild animals in several different environments [10, 19, 21, 29, 30].

Our two cases of *Bartonella henselae* infection exemplify two difficulties in defining *Bartonella* IE. Firstly, finding the vegetation in the valvular endocardium in the echocardiography proved to be very difficult. Secondly, although both patients had a subacute temporal course with weight loss and changes in kidney function, they did not have vascular phenomena. *Bartonella* endocarditis is a diagnostic challenge even for cardiac surgery referral centers. Health professionals should be alert to an atypical case of infective endocarditis, especially when patients have weight loss, kidney injury, and epidemiological history for domestic animals with ectoparasites.

## Supplementary Information

**Additional file 1:** **Methods S1.** Serological and molecular investigation. **Table S1.** Serological results of serum samples of the patients and their pets. **Table S2.** *Bartonella* species detected in paraffin-fixed human tissue and blood samples from Patients P1 and P2.

## Data Availability

The data that support the findings of the current study are available from the corresponding author upon reasonable request. All sequences generated in this study are available in Genbank (MZ666121–MZ666124).

## Abbreviations

BCNIE: Blood culture-negative infective endocarditis
IE: Infective endocarditis
HACEK: *Haemophilus* spp., *Aggregatibacter* spp., *Cardiobacterium hominis*, *Eikenella corrodens*, and *Kingella* spp.
IgG: Immunoglobulin type G
HUPE-UERJ: Pedro Ernesto University Hospital of State of Rio de Janeiro
CRP: C-reactive protein
ESR: Erythrocyte sedimentation rate
RF: Rheumatoid factor
IFA: Indirect immunofluorescence assay
PCR: Polymerase chain reaction
NRLR: National Reference Laboratory for Rickettsioses
IOC: Oswaldo Cruz Institute
FIOCRUZ: Oswaldo Cruz Foundation
DNA: Deoxyribonucleic acid
BLAST: Basic local alignment search tool
CSD: Cat scratch disease
