# Supplementary material for: Infectious endocarditis caused by Bartonella henselae associated with infected pets: two case reports
Source: J Med Case Rep. 2023 Apr 19;17:143. doi: 10.1186/s13256-023-03839-8 (PMC10112992; doi:10.1186/s13256-023-03839-8)
Supplement: Supplementary file 1 — Additional file 1: Methods S1. Serological and molecular investigation. Table S1. Serological results of serum samples of the patients and their pets. Table S2. Bartonella species detected in paraffin-fixed human tissue and blood samples from Patients P1 and P2. [file 13256_2023_3839_MOESM1_ESM.docx]

**Methods S1 Serological and molecular investigation**

Serum samples from patients and their pets were evaluated by IFA for IgG antibodies against *Bartonella* spp. using the commercial Kit Scimedx/Medivax® with a titer cut-off of 64, following the manufacturer’s instructions.

Total blood and serum samples and two paraffin-fixed tissue samples were submitted to extraction of total DNA and polymerase chain reaction (PCR) with specific primers to identify *Bartonella* spp*.* DNA*.* The extraction of total DNA was done using a commercial kit (QIAamp DNA Mini Kit, Qiagen®) according to the manufacturer’s instructions and the extracted DNA quality was performed using agarose gels. These DNA samples were tested by conventional PCR for the presence of *Bartonella* spp. *Bartonella* DNA detected in human samples and nuclease free water (UltraPure™ DNase/RNase-Free – Invitrogen) was used as the positive and negative control in molecular tests. DNA samples were submitted to conventional PCR for *gltA*(~700bp –Rozental *et al.* 2014), *groEL* (~700bp – Gonçalves-Oliveira *et al.* 2020) and *htrA* (~400bp - Anderson *et al.* 1994). The assays of *gltA* and *groEL* were run for each gene containing 0.5 μL of each primer (10mM), 0.5 μL of 20 mM dNTP (Invitrogen™), 4.0 μL of 50 mM MgCl_2_ (Applied Biosystems^®^), 2.5 μL of 10x PCR buffer (Applied Biosystems^®^), 0.2 μL of AmpliTaq Gold® DNA Polymerase (5U/μl, Applied Biosystems^®^), 13.8 μL of nuclease-free water (UltraPure™ DNase/RNase-Free –Invitrogen) and 3 μL of the sample DNA in a final volume of 25 μL. The cycle conditions of *gltA* were initial denaturation at 95ºC for 10 min, 35 cycles of denaturation at 95ºC for 30s, annealing at 58ºC for 30s and extension of 72ºC for 45s, and final extension of 72ºC for 8 min. The cycle conditions of *groEL* were initial denaturation at 95ºC for 10 min, 35 cycles of denaturation at 95ºC for 30s, annealing at 54ºC for 30s, and extension of 72ºC for 45s, and final extension of 72ºC for 8 min. The assay of *htrA* was performed for each gene containing 0.6μL of each primer (10mM), 0.25μL of dNTP 20mM (Invitrogen™), 0.75μL of MgCl_2_ 50mM (Invitrogen™), 2.5μL of Buffer 10X PCR (Invitrogen™), 0.1μL of Taq Platinum™ DNA Polymerase, 16.2μL of nuclease free water (UltraPure™ DNase/RNase-Free – Invitrogen) and 4μL of DNA sampling, on a mixture with a final volume of 25 μL. The cycle conditions of *htrA* were initial denaturation at 94ºC for 5 minutes, 35 cycles of denaturation at 94ºC for 45s, annealing at 56ºC for 45s and extension of 72ºC for 45s, and a final extension at 72ºC for 7 minutes. For the sequencing reaction, the amplified products were purified using Illustra GFX PCR DNA and Gel Band Purification Kit (GE Healthcare ©) and were sequenced using the BigDye Terminator Cycle Sequencing Ready Reaction®v3.1kit (Thermo Fisher Scientific™, Waltham, MA, USA). The nucleotide sequences, their chromatograms, the consensus sequence, and the divergence in the nucleotides between sequences, were all elucidated by a contig analysis run in Geneious^®^11.1.5 [32]*.* The comparisons with sequences deposited in Genbank were made using the basic local alignment search tool (BLAST).

**Table S1** Serological results of serum samples of the patients and their pets

| **Samples** | **Type** | **Collect 1** | **Titers of IgG-antibodies**  ***Bartonella* spp.** | **Collect 2** | **Titers of IgG-antibodies**  ***Bartonella* spp.** | **Collect 3** | **Titers of IgG-antibodies**  ***Bartonella* spp.** | **Collect 4** | **Titers of IgG-antibodies**  ***Bartonella* spp.** |
| --- | --- | --- | --- | --- | --- | --- | --- | --- | --- |
| **Patient P1** | Serum | 9/Jul/20 | 8192 | 23/Jul/20 | 16384 | 16/Sep/20 | 32768 | 25/Sep/20 | 2048 |
| **Wife’s Patient P1** | Serum | 9/Jul/20 | Not reactive | - |  | - |  | - |  |
| **Dog 1– Patient P1** | Serum | - |  | - |  | - |  | 25/Sep/20 | ≥ 128 |
| **Dog 2– Patient P1** | Serum | - |  | - |  | - |  | 25/Sep/20 | Not reactive |
| **Patient P2** | Serum | 23/Dez/20 | 2048 | 07/Jan/21 | 512 | - |  | - |  |
| **Cat 1 – Patient P2** | Serum | - |  | - |  | 09/Fev/21 | 128 |  |  |
| **Cat 2 – Patient P2** | Serum | - |  | - |  | 09/Fev/21 | 128 |  |  |
| **Cat 3 – Patient P2** | Serum | - |  | - |  | 09/Fev/21 | 128 |  |  |

**Table S2** *Bartonella* species detected in paraffin-fixed human tissue and blood samples from Patients P1 and P2

| **Samples** | ***Bartonella* species**^†^ | **Loci ID‡**  **(Size bp)** | **Similarities**  **(% reference strain)** | **Genbank accession number** |
| --- | --- | --- | --- | --- |
| Patient P1 | *B. henselae* | *gltA*(723bp) | 99.84 % - KX024515 - Uncultured Bartonella of domestic cat blood samples (*B. henselae*) | MZ666124 |
|  |  | *groEL*(747bp) | 100% HQ704721 - *Bartonella henselae* isolate of feral cat blood | MZ666123 |
|  |  | *htrA*(409bp) | 100% CP020742 - *Bartonella henselae* strain Houston-I | MZ666122 |
| Patient P2 | *B. henselae* | *htrA*(421bp) | 99.76 % CP020742 - *Bartonella henselae* strain Houston-I | MZ666121 |
